# Integrating intratumoral, peritumoral, and clinical features in an ultrasound-based radiomics model: contributions and synergies for predicting microvascular invasion in hepatocellular carcinoma

**DOI:** 10.3389/fonc.2025.1566105

**Published:** 2025-09-01

**Authors:** Hong Fu, Yanhua Huang, Baochun Lu, Jianhua Yu, Difan Zhou, Chuanling Hou, Luohang Xu, Hongwei Qian

**Affiliations:** ^1^ Department of Hepatobiliary and Pancreatic Surgery, Shaoxing People’s Hospital, Shaoxing, China; ^2^ Shaoxing Key Laboratory of Minimally Invasive Abdominal Surgery and Precise Treatment of Tumor, Shaoxing, China; ^3^ Department of Ultrasound, Shaoxing People’s Hospital, Shaoxing, China; ^4^ Department of Pathology, Shaoxing People’s Hospital, Shaoxing, China; ^5^ School of Medicine, Shaoxing University, Shaoxing, Zhejiang, China

**Keywords:** hepatocellular carcinoma, microvascular invasion, ultrasound radiomics, intratumoral and peritumoral features, logistic regression, predictive modeling

## Abstract

**Background:**

Microvascular invasion (MVI) is a critical determinant of poor prognosis in hepatocellular carcinoma (HCC). Accurate preoperative prediction of MVI is essential for optimizing surgical and therapeutic strategies. This study aims to develop a combined model integrating intratumoral, peritumoral, and clinical features from ultrasound-based radiomics for MVI prediction.

**Methods:**

Ultrasound images of 119 patients with pathologically confirmed HCC were analyzed. A total of 1,414 radiomics features were extracted from intratumoral and peritumoral regions. Feature selection was performed using intraclass correlation coefficient (ICC) analysis, t-tests, and least absolute shrinkage and selection operator (LASSO) regression. Logistic regression, Random Forest, and other machine learning algorithms were applied to construct predictive models. The best-performing intratumoral, peritumoral, and clinical models were combined using logistic regression. SHapley Additive exPlanations (SHAP) analysis, logistic regression coefficients, and partial dependence analysis were employed to evaluate feature contributions and interactions.

**Results:**

Both intratumoral and peritumoral models achieved high AUCs (0.781 and 0.792, respectively), with no statistically significant difference between them. The combined model, incorporating tumor size, achieved the highest AUC (0.903, 95% CI: 0.780–1.000) and superior performance across all evaluation metrics. Tumor size exhibited the smallest logistic regression coefficient but the highest SHAP contribution, indicating strong interactions with intratumoral and peritumoral features. Interaction analyses revealed that the combined effects of tumor size and radiomics features significantly enhanced predictive performance.

**Conclusion:**

This study demonstrates that combining intratumoral, peritumoral, and clinical features enhances the predictive accuracy for MVI in HCC. The findings underscore the value of feature integration and interactions, providing insights for personalized treatment planning and advancing the clinical utility of ultrasound-based radiomics.

## 1 Introduction

Hepatocellular carcinoma (HCC) is a leading cause of cancer-related mortality worldwide (1, 2). Microvascular invasion (MVI) is a critical pathological feature of HCC, significantly associated with aggressive tumor behavior, early recurrence, and poor prognosis (3). Accurate preoperative prediction of MVI is crucial for guiding clinical decision-making, including determining surgical margins, the necessity of adjuvant therapies, and personalized treatment strategies (4, 5). However, predicting MVI preoperatively remains challenging with existing imaging modalities such as computed tomography (CT) and magnetic resonance imaging (MRI), which have limitations in detecting microscopic tumor invasion (6–8).

Radiomics, a rapidly evolving field in medical imaging, offers a non-invasive method to extract high-dimensional quantitative features from medical images, enabling deeper insights into tumor heterogeneity (9, 10). While CT- and MRI-based radiomics models have demonstrated promising results in predicting MVI (11, 12), the application of radiomics in ultrasound (US) imaging remains underexplored. Ultrasound imaging, with its real-time capabilities, non-invasive nature, and lower cost, is a particularly appealing modality for radiomics research, especially in regions with limited access to advanced imaging techniques. Integrating both intratumoral and peritumoral regions in radiomics analysis may provide a more comprehensive characterization of MVI-related features, as peritumoral tissue often contains crucial information about tumor invasiveness and interactions with the surrounding microenvironment (13–16).

Despite these advances, several gaps remain. First, ultrasound-based radiomics is underutilized compared to CT or MRI, despite the unique advantages of US imaging. Second, many existing studies focus exclusively on intratumoral features, neglecting the valuable predictive information contained in the peritumoral region. Third, while combining intratumoral, peritumoral, and clinical parameters in a single model shows promise, the individual contributions and interactions of these components within a combined model remain unclear.

Our study aims to address these gaps by developing a radiomics model based on preoperative ultrasound imaging that integrates both intratumoral and peritumoral features. Additionally, a combined model will be constructed by integrating radiomics features with clinical characteristics using logistic regression to improve predictive performance. Furthermore, interpretable analysis methods will be incorporated to visualize and explain the contributions of specific radiomics and clinical features, enhancing the model’s transparency and clinical applicability.

## 2 Material and methods

### 2.1 Study population

This retrospective study was conducted at Shaoxing People’s Hospital, following approval from the Institutional Review Board. Written informed consent was obtained from all patients prior to participation.

A total of 119 patients with pathologically confirmed HCC who underwent surgical resection at Shaoxing People’s Hospital between September 2019 and May 2024 were enrolled. Inclusion criteria were as follows: (1) preoperative ultrasound imaging performed within two weeks before surgery; (2) availability of complete clinical and pathological data; (3) no prior treatment such as transarterial chemoembolization (TACE) or radiofrequency ablation (RFA) before surgery; and (4) pathological confirmation of HCC with or without MVI. Exclusion criteria included poor-quality ultrasound images unsuitable for radiomics analysis (n = 9), patients with other concurrent malignancies (n = 16), and incomplete clinical data (n = 45).

The study cohort was divided into training and validation sets at a 7:3 ratio for model development and evaluation. Baseline characteristics, including demographic, clinical, and pathological data, were collected from medical records (**Table 1**). Microvascular invasion was defined as the presence of tumor cells within a vascular space lined by endothelial cells beyond the tumor boundary, as confirmed by histopathological examination.

**Table 1:** Demographic and clinical characteristics of patients.

Variables	MVI negative(n=83)	MVI positive(n=36)	P	Training group(n=83)		P	Testing group(n=36)		P
				MVI negative(n=59)	MVI positive(n=24)		MVI negative(n=24)	MVI positive(n=12)	
Age(year)	65.39 ± 9.43	63.72 ± 13.05	0.44	66.51 ± 9.64	64.25 ± 12.16	0.38	63.5(56.5-68.0)	66.0(51.0-74.5)	0.993
AFP(mg/mL)	9.62(2.84-60.74)	36.64(7.56-983.68)	0.133	156.87 ± 426.8	1763.32 ± 7088.25	0.091	1602.85 ± 6897.89	9891.91 ± 22613.71	0.118
ALT(IU/L)	36.77 ± 32.95	45.08 ± 44.99	0.267	38.42 ± 36.14	48.45 ± 51.53	0.324	32.71 ± 22.82	38.35 ± 26.35	0.524
AST(IU/L)	41.4 ± 34.11	52.24 ± 59.32	0.216	44.79 ± 38.76	57.97 ± 70.06	0.284	33.08 ± 15.23	40.79 ± 23.33	0.255
TBIL(µmol/L)	16.68 ± 9.73	19.63 ± 26.22	0.378	16.24 ± 8.6	16.85 ± 13.9	0.811	17.77 ± 11.99	25.18 ± 40.37	0.426
DBIL(µmol/L)	5.35 ± 4.19	8.38 ± 15.52	0.106	5.41 ± 4.45	6.48 ± 6.67	0.404	5.2 ± 3.44	12.17 ± 24.73	0.197
ALB (g/L)	38.81 ± 4.24	38.72 ± 4.36	0.92	38.66 ± 4.15	38.32 ± 4.49	0.743	39.18 ± 4.45	39.53 ± 3.96	0.822
PT(s)	13.08 ± 1.31	13.21 ± 1.44	0.633	12.97 ± 1.01	13.16 ± 1.47	0.5	13.35 ± 1.82	13.3 ± 1.38	0.941
INR	1.05 ± 0.13	1.06 ± 0.12	0.798	1.04 ± 0.09	1.06 ± 0.12	0.291	1.08 ± 0.18	1.04 ± 0.11	0.517
Tumor Size	4.0(2.5-5.25)	5.3(4.0-8.6)	0.002^*^	4.29 ± 2.47	5.87 ± 3.12	0.019^*^	4.28 ± 2.19	7.18 ± 3.25	0.004^*^
Sex			0.13			0.103			0.733
Female	15	11		12	9		3	2	
Male	68	25		47	15		21	10	
HBsAg			0.63			0.801			0.599
Negative	24	12		18	8		6	4	
Positive	59	24		41	16		18	8	
Cirrhosis			0.981			0.841			0.813
Absent	39	17		26	10		13	7	
Present	44	19		33	14		11	5	
Multifocality			0.056			0.135			0.257
Absent	72	26		52	18		20	8	
Present	11	10		7	6		4	4	

AFP, alpha fetoprotein; ALB, albumin level; ALT, alanine aminotransferase; AST, aspartate aminotransferase; TBIL, total bilirubin; DBIL, directed bilirubin; PT, prothrombin time; INR, international normalized ratio; *p<0.05.

**Figure 1:**
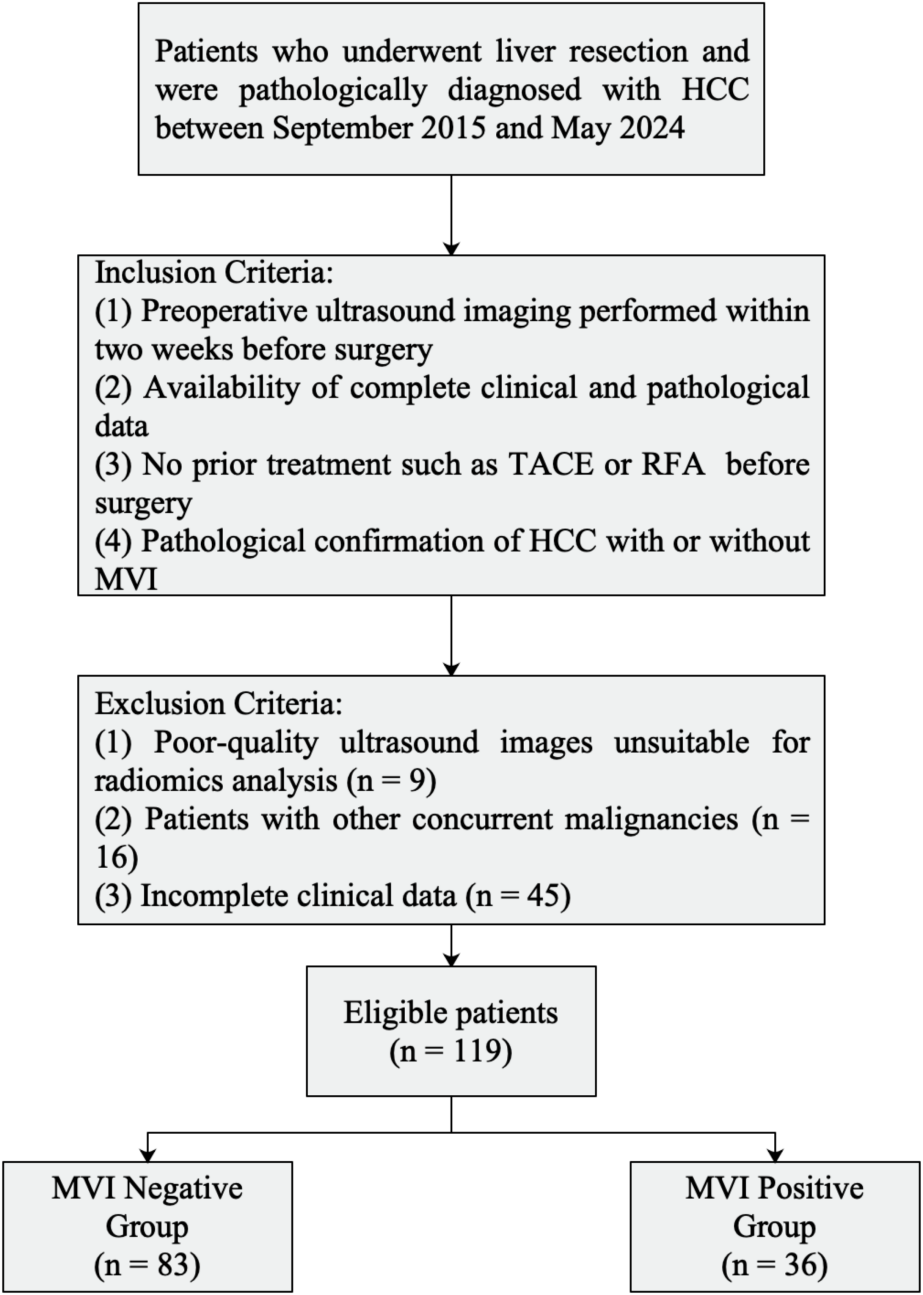
Flowchart of included and excluded patients.

A flowchart illustrating the inclusion and exclusion of patients is presented in **Figure 1**.

### 2.2 Ultrasound procedure

Preoperative gray-scale ultrasound imaging was performed using various ultrasound machines (details provided in the **Supplementary Materials**). All patients underwent standardized ultrasound examination by experienced radiologists two weeks prior to surgery.

During the examination, patients were positioned in a supine or left lateral decubitus position to optimize visualization of the liver and associated structures. The scanning protocol included assessing tumor location, size, echogenicity, and boundary characteristics. Particular attention was given to the peritumoral region to ensure comprehensive imaging data were collected for subsequent radiomics analysis.

Images were stored in Digital Imaging and Communications in Medicine (DICOM) format for consistency and compatibility with radiomics feature extraction workflows.

### 2.3 Histological and immunohistochemistry

Histopathological examination was performed on surgically resected HCC specimens to confirm the diagnosis and assess MVI. Tissue samples were fixed in 10% formalin, embedded in paraffin, and sectioned at 4-µm thickness. Hematoxylin and eosin staining was used to evaluate tumor differentiation and the presence of MVI. Microvascular invasion was defined as the presence of tumor cells within a vascular lumen lined by endothelial cells beyond the tumor border. Representative HE-stained histological images illustrating MVI-negative (M0), MVI-positive (M1 and M2) are shown in **Supplementary Figure S1**.

### 2.4 Region of interest delineation

**Figure 2:**
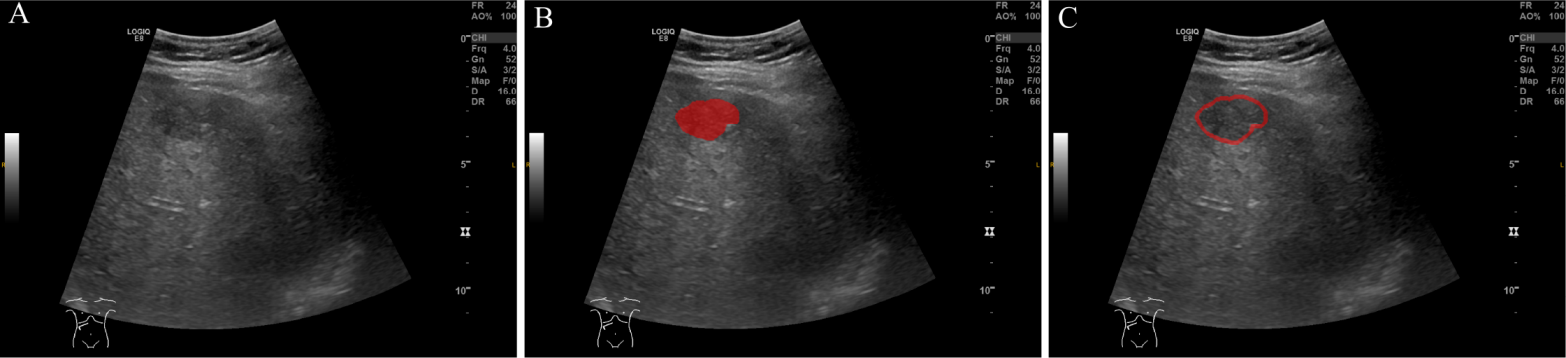
ROI (Region of Interest) delineation on ultrasound images. **(A)** Original ultrasound image. **(B)** ROI delineation of the tumor region (in red). **(C)** ROI delineation including the peritumoral region (in red), encompassing the area surrounding the tumor.

Region of interest (ROI) delineation was performed using ITK-SNAP software(Version 4.0.0, www.itksnap.org) (17) (**Figure 2**). Initially, the tumor region was manually delineated by two independent ultrasound radiologists. For peritumoral analysis, the tumor ROI was automatically expanded outward by 1 cm, creating a peritumoral region. This distance was selected based on prior study suggesting that a 1 cm peritumoral zone is most suitable for MVI prediction, as it effectively captures both local tumor invasion and critical tissue interactions near the tumor boundary (18). These automatically generated ROIs were manually adjusted to ensure accuracy and exclude irrelevant structures such as large vessels or adjacent organs. To minimize bias, the two radiologists independently performed ROI delineation with a 1-week interval between assessments and were blinded to the clinical and imaging data.

### 2.5 Radiomics feature extraction and dimension reduction

Radiomics feature extraction was performed on normalized ultrasound images to ensure consistency across imaging systems. The normalization process included resampling images to a uniform spatial resolution of 3 × 3 × 3 mm³, scaling intensity values to 32 gray levels based on a scale of 255, and suppressing machine-specific artifacts or noise. PyRadiomics software was utilized to extract features from both intratumoral and peritumoral regions. After feature extraction, the features were standardized by applying z-score normalization to ensure that all features were on a comparable scale, improving the stability and performance of subsequent analyses. These features encompassed shape characteristics, such as volume and compactness, first-order statistics to describe intensity distributions, texture metrics derived from gray-level co-occurrence and size zone matrices, and higher-order features from wavelet decompositions.

To reduce dimensionality and improve model robustness, a systematic feature selection process was applied. Features with low reproducibility, as assessed by intra- and inter- correlation coefficient (ICC), were excluded (threshold: ICC < 0.75). Highly correlated features (correlation coefficient > 0.8) were removed to eliminate redundancy. Independent samples t-tests were conducted to identify features significantly associated with MVI (p < 0.05), and least absolute shrinkage and selection operator (LASSO) regression was applied to refine the feature set further, prioritizing the most informative predictors for subsequent model development.

### 2.6 Model construction

Separate models for intratumoral and peritumoral features were developed using various modeling techniques, all trained on the same subset of features selected via LASSO for each region. Optimal hyperparameters were determined using a two-step approach combining Random Search and Grid Search with cross-validation. The best-performing intratumoral and peritumoral models were then combined using logistic regression, incorporating clinical parameters to construct a comprehensive model.

Model performance was assessed using metrics, including AUC, sensitivity, specificity, and accuracy et al. To account for the relatively small sample size, bootstrapping with 1,000 resamples was performed to estimate the confidence intervals of model metrics and to compare the predictive performance of different models statistically. Model evaluation and comparisons were conducted on the validation dataset to ensure robustness and generalizability.

### 2.7 Analysis of feature importance in the combined model

To analyze the contributions of intratumoral and peritumoral models, along with clinical parameters, within the combined framework, several interpretability techniques were employed. SHAP (Shapley Additive Explanations) analysis quantified both global and local feature importance by perturbing feature values and observing the resulting changes in model predictions. Logistic regression coefficients provided a linear quantification of feature contributions, reflecting the direction and magnitude of each feature’s impact on classification outcomes. Partial dependence analysis (PDA) was conducted to visualize how variations in individual feature values influenced model predictions, highlighting both independent contributions and interactions.

### 2.8 Statistical analysis

All statistical analyses were performed using Python software (Version 3.11). Continuous variables were expressed as either mean ± standard deviation for normally distributed data or median with the range for non-normally distributed data. Comparative analyses of continuous variables were conducted using the independent samples t-test for normally distributed data or the Mann-Whitney U test for non-normally distributed data. Categorical variables were reported as frequencies or percentages and analyzed using chi-square tests or Fisher’s exact tests, as appropriate. Comparisons of ROC curves were performed using a bootstrapping method with 1,000 resamples to estimate confidence intervals and evaluate the differences in predictive performance. A two-tailed p-value of <0.05 was considered statistically significant throughout the analysis.

## 3 Results

### 3.1 Characteristics of the study population

A total of 119 patients were included in this study, comprising 83 MVI-negative and 36 MVI-positive cases. The baseline characteristics of the study population are summarized in **Table 1**. Tumor size was significantly larger in the MVI-positive group compared to the MVI-negative group across the entire cohort (median [IQR]: 4.0 [2.5–5.25] cm vs. 5.3 [4.0–8.6] cm; p < 0.05). This difference remained significant in both the training cohort (4.29 ± 2.47 cm vs. 5.87 ± 3.12 cm; p = 0.019) and the testing cohort (4.28 ± 2.19 cm vs. 7.18 ± 3.25 cm; p = 0.004).

Serum alpha-fetoprotein (AFP) levels were higher in the MVI-positive group compared to the MVI-negative group, though the difference was not statistically significant (p = 0.133). Other baseline characteristics, including age, liver function markers, and sex, showed no statistically significant differences between the two groups (all p > 0.05). And all the clinical characteristics of the training and testing cohorts showed no significant differences (**Supplementary Table S1**).

### 3.2 Feature selection

A total of 1,414 radiomics features were extracted from both intratumoral and peritumoral regions. Due to the limited number of MVI-positive samples, the Synthetic Minority Oversampling Technique (SMOTE) was applied to balance the data within the training set.

Feature selection involved several steps: ICC analysis to ensure feature reproducibility. Both within-group (intraclass) and between-group (interclass) correlation coefficients were calculated. Features with an ICC value less than 0.75 for either within-group or between-group consistency were excluded to maintain high reproducibility between observers and across groups. In the intratumoral region, 1373 of features were retained after ICC analysis, while 1352 were retained for the peritumoral region. Removal of collinear features addressed redundancy, t-tests were used for univariate analysis, and LASSO regression was applied for dimensionality reduction (details of the LASSO process are provided in **Supplementary Figures S2, S3**). As a result, 8 radiomics features were selected for the intratumoral model and 6 features for the peritumoral model. The selected features are presented in **Table 2**.

**Table 2:** The LASSO selected features and their coefficients.

Model	Filter	Feature class	Feature	Coefficient
Intratumoral Model	diagnostics	Mask-original	VoxelNum	-0.076064
	original	glcm	Correlation	0.068984
	wavelet-LLH	glrlm	ShortRunHighGrayLevelEmphasis	0.027603
	wavelet-LLH	glszm	SizeZoneNonUniformityNormalized	0.074378
	wavelet-LHH	glrlm	GrayLevelNonUniformityNormalized	-0.062907
	wavelet-HLH	glszm	SizeZoneNonUniformityNormalized	-0.028195
	wavelet-HHH	glszm	SizeZoneNonUniformityNormalized	0.110769
	square	ngtdm	Busyness	0.12071
Peritumoral Model	wavelet-LHL	glcm	Imc1	0.06622
	wavelet-LHH	glszm	SmallAreaEmphasis	0.098223
	wavelet-HLL	glszm	ZoneEntropy	0.046182
	wavelet-HLH	glszm	ZoneEntropy	0.000931
	wavelet-HHL	glcm	InverseVariance	0.151837
	square	ngtdm	Coarseness	-0.045678

### 3.3 Model construction

**Figure 3:**
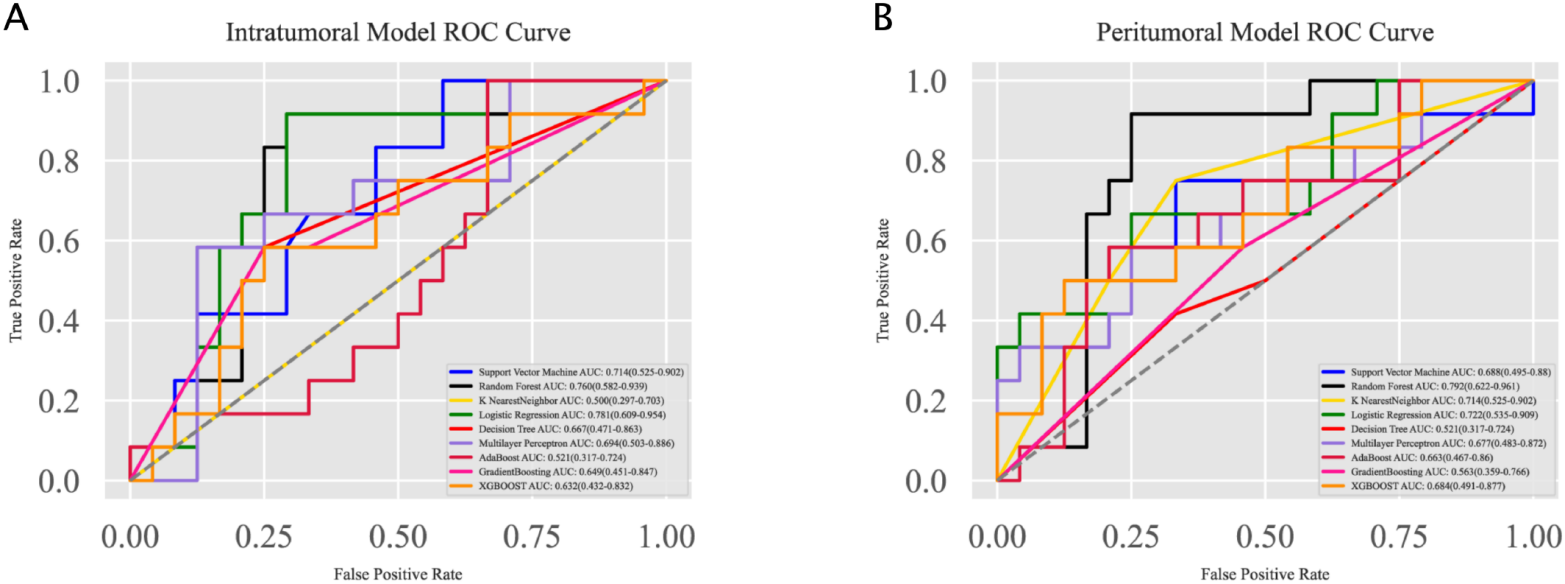
Receiver Operating Characteristic (ROC) curves for the intratumoral and peritumoral models. **(A)** ROC curves for the intratumoral model constructed using various machine learning algorithms. Logistic Regression achieved the highest AUC of 0.781, followed by Random Forest with an AUC of 0.760. **(B)** ROC curves for the peritumoral model constructed using the same algorithms. Random Forest achieved the highest AUC of 0.792, followed by Logistic Regression with an AUC of 0.722.

To construct predictive models, multiple machine learning algorithms were employed, including Support Vector Machine, Random Forest, K Nearest Neighbor, Logistic Regression, Decision Tree, Artificial Neural Network, AdaBoostClassifier, GradientBoostingClassifier, and XGBOOST. The ROC curves for different modeling methods are summarized in **Figures 3A, B** for the intratumoral and peritumoral models, respectively. Hyperparameter optimization was performed using a two-step approach combining RandomizedSearchCV and GridSearchCV with five-fold cross-validation. The detailed search ranges and the final selected parameters for each model are provided in the **Supplementary Materials** (**Supplementary Tables S2–S4**).

For the intratumoral model, Logistic Regression achieved the highest AUC of 0.781 (95% CI: 0.609–0.954), followed by Random Forest with an AUC of 0.760 (95% CI: 0.582–0.939). For the peritumoral model, Random Forest performed best with an AUC of 0.792 (95% CI: 0.622–0.961), while Logistic Regression also showed strong performance with an AUC of 0.722 (95% CI: 0.535–0.909).

**Figure 4:**
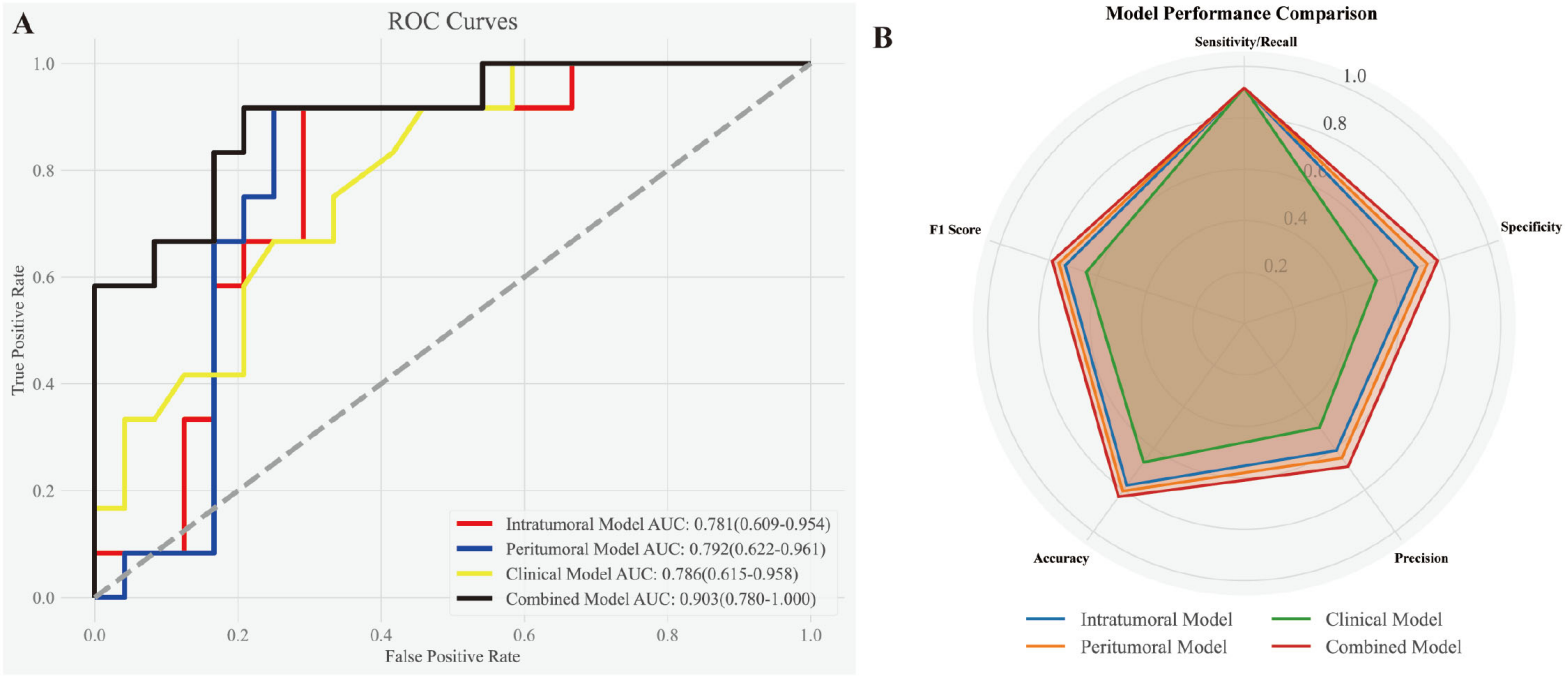
Performance evaluation of the intratumoral, peritumoral, clinical, and combined models. **(A)** ROC curves demonstrating the discriminatory power of the models for predicting MVI status. The combined model exhibited the highest AUC (0.903, 95% CI: 0.780–1.000), outperforming the clinical model (AUC: 0.786, 95% CI: 0.615–0.958), peritumoral model (AUC: 0.792, 95% CI: 0.622–0.961), and intratumoral model (AUC: 0.781, 95% CI: 0.609–0.954). **(B)** Radar chart illustrating various evaluation metrics, including sensitivity/recall, specificity, precision, accuracy, and F1 score, for the four models. The combined model consistently achieved the best overall performance across all metrics.

The best-performing intratumoral and peritumoral models (Logistic Regression and Random Forest, respectively) were combined with the clinical parameter tumor size using a logistic regression approach to construct the combined model. The performance evaluation is presented in **Figure 4**. (A) The ROC curves highlight the discriminatory power of the models for predicting MVI status. The combined model achieved the highest AUC (0.903, 95% CI: 0.780–1.000), surpassing the clinical model (AUC: 0.786, 95% CI: 0.615–0.958), the peritumoral model (AUC: 0.792, 95% CI: 0.622–0.961), and the intratumoral model (AUC: 0.781, 95% CI: 0.609–0.954). (B) The radar chart provides a visual comparison of evaluation metrics, including sensitivity/recall, specificity, precision, accuracy, and F1 score, demonstrating the combined model’s superior overall performance and robustness.

**Table 3** summarizes the performance metrics of all four models. While all models achieved the same sensitivity (0.917), the combined model outperformed the others in accuracy (0.833), specificity (0.792), precision (0.688), and F1 score (0.786), reflecting its better balance between precision and recall. The peritumoral model showed slightly better performance than the intratumoral model, with higher specificity (0.75 vs. 0.708), precision (0.647 vs. 0.611), and F1 score (0.759 vs. 0.733). By contrast, the clinical model (tumor size) demonstrated the lowest specificity (0.542) and precision (0.5), indicating its limitations as a standalone predictor. These results further emphasize the strength of the combined model in achieving superior diagnostic performance.

**Table 3:** The performance of the four models in predicting MVI.

Evaluation indicators	Intratumoral model	Peritumoral model	Tumor size	Combined model
tp	11	11	11	11
tn	17	18	13	19
fp	7	6	11	5
fn	1	1	1	1
Sensitivity	0.917	0.917	0.917	0.917
Specificity	0.708	0.75	0.542	0.792
Precision	0.611	0.647	0.5	0.688
Recall	0.917	0.917	0.917	0.917
Accuracy	0.778	0.806	0.667	0.833
F1 Score	0.733	0.759	0.647	0.786
AUC	0.781	0.792	0.786	0.903

tp, true positive; fp, false positive; fn, false negative; tn, true negative; AUC, area under the curve.

To statistically compare the performance of the models, bootstrapping was performed. The results showed no significant differences between the intratumoral model and the combined model (p = 0.184) or between the peritumoral model and the combined model (p = 0.124). However, a significant difference was observed between the clinical model and the combined model (p = 0.026), highlighting the added predictive value of integrating radiomics features with clinical parameters. Comparisons between the intratumoral and peritumoral models (p = 0.952) and between the clinical model and both the intratumoral (p = 0.978) and peritumoral models (p = 0.982) revealed no significant differences.

In addition to discrimination performance, we evaluated the probabilistic accuracy of the combined model. The calibration curve showed overall good agreement between predicted and observed probabilities, with only mild deviations observed in the mid-probability range (**Supplementary Figure S4**). The Brier score was 0.151, indicating acceptable probabilistic accuracy. The Hosmer–Lemeshow goodness-of-fit test was non-significant (χ² = 11.45, p = 0.178), suggesting no significant miscalibration.

### 3.4 Analysis of feature contributions in the combined model

**Figure 5:**
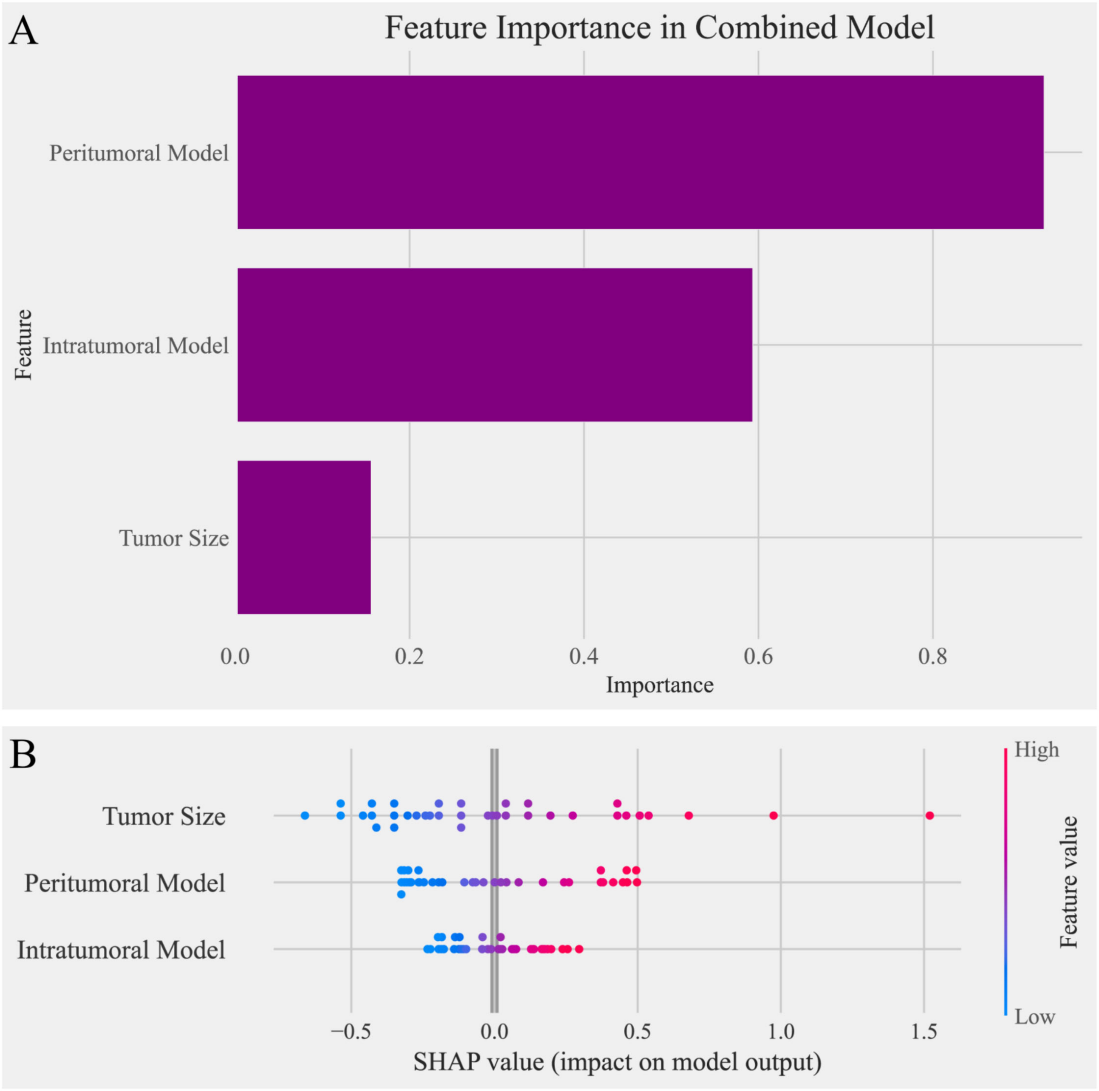
Feature importance analysis in the combined model. **(A)** Bar chart of feature importance based on logistic regression coefficients in the combined model. The peritumoral model contributed the most to the predictions, followed by the intratumoral model, while tumor size showed the lowest importance in this analysis. **(B)** SHAP (Shapley Additive Explanations) plot illustrating the impact of individual features on the combined model’s predictions. Tumor size exhibited the highest SHAP values, indicating its dominant contribution to the model’s output, followed by the peritumoral model and intratumoral model. The contrasting results between the two analyses highlight the differences in feature importance interpretations.

To analyze the contributions of individual features within the combined model, several interpretability techniques were employed. Logistic regression coefficients were analyzed to evaluate the linear contribution of each feature, with results presented in **Figure 5A**. Tumor size showed the lowest importance in this analysis. SHAP analysis was then used to quantify the global and local importance of each feature, as shown in **Figure 5B**. In contrast to the logistic regression analysis, tumor size demonstrated the highest contribution in the SHAP analysis, highlighting its critical role in the combined model’s predictions.

**Figure 6:**
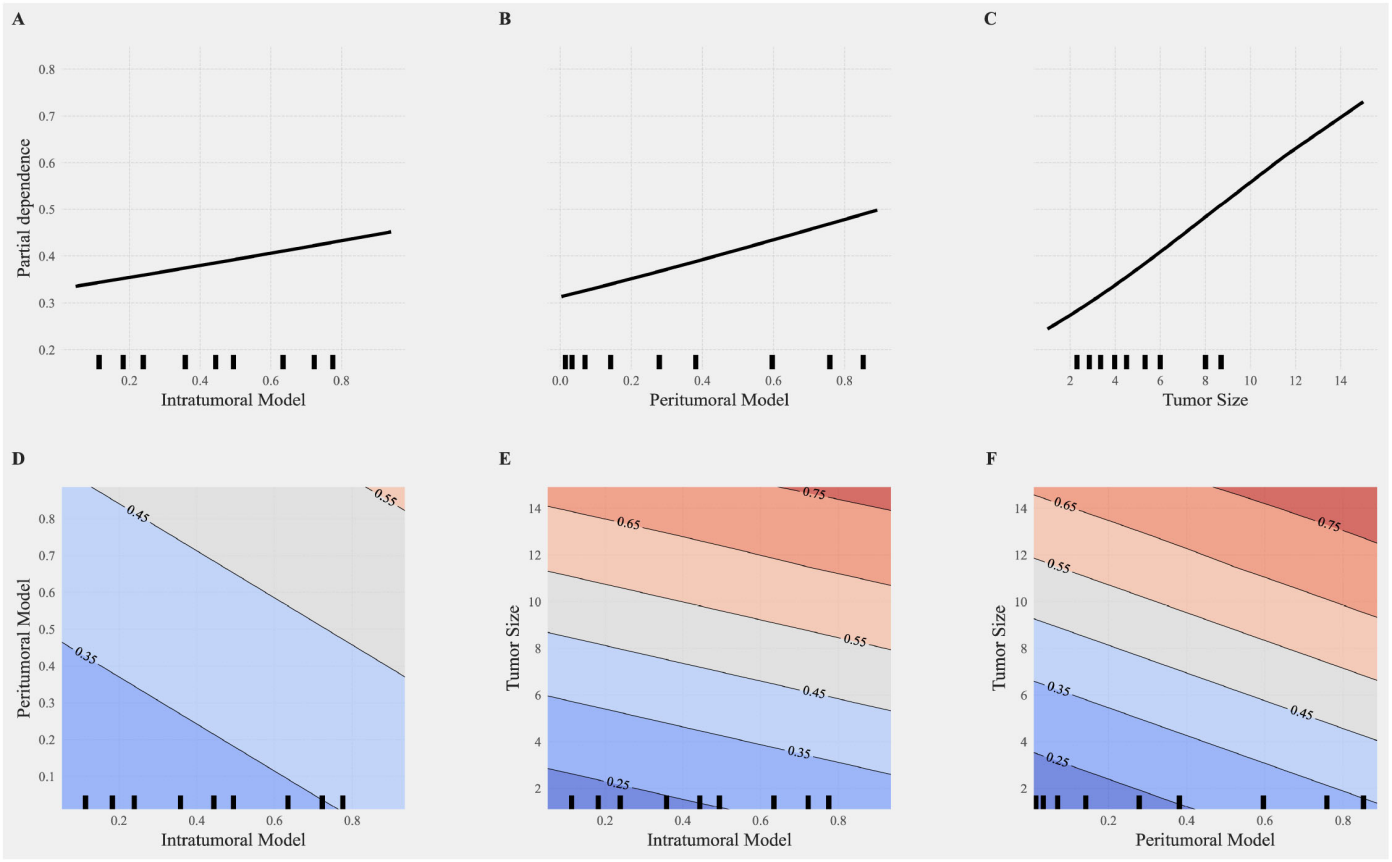
Partial dependence analysis of individual and interactive features in the combined model. **(A–C)** Partial dependence plots (PDP) for single features, including the intratumoral model **(A)**, peritumoral model **(B)**, and tumor size **(C)**. The X-axis represents feature values, and the Y-axis represents partial dependence values, indicating the positive or negative impact of each feature on the model predictions. Among these, tumor size **(C)** shows the most significant increase, highlighting its critical importance in the prediction outcomes. **(D–F)** Interaction effects between features visualized through 2D partial dependence heatmaps. **(D)** Interaction between intratumoral and peritumoral models. **(E)** Interaction between tumor size and the intratumoral model. **(F)** Interaction between tumor size and the peritumoral model. The heatmaps illustrate the contributions of these interactions to the combined model, with more pronounced color gradients indicating stronger interaction effects on the model predictions. High values of tumor size combined with radiomics features (intratumoral or peritumoral) significantly enhance the probability of positive class predictions.

PDA was further applied to evaluate the impact of individual features and their interactions on the model’s predictions. **Figures 6A–C** illustrate the partial dependence plots for the intratumoral model, peritumoral model, and tumor size, respectively. The X-axis represents feature values, and the Y-axis represents partial dependence values, reflecting the positive or negative influence of each feature on the predicted probability. Among these, tumor size (**Figure 6C**) exhibited the most significant linear increase, emphasizing its critical role in prediction outcomes.

**Figures 6D–F** depict the interaction effects between features using 2D partial dependence heatmaps. The interactions between the intratumoral and peritumoral models (**Figure 6D**), tumor size and the intratumoral model (**Figure 6E**), and tumor size and the peritumoral model (**Figure 6F**) reveal substantial contributions to the combined model. The pronounced color gradients in these heatmaps indicate that stronger interactions significantly enhance the model’s predictive performance. High values of tumor size combined with radiomics features result in a marked increase in the probability of positive class predictions, underscoring the synergistic effects of these interactions in improving model performance. The PDA results revealed that tumor size and the peritumoral model exhibited stronger independent contributions to positive class predictions, showing significant linear growth trends. In contrast, the intratumoral model’s independent contribution was weaker but still showed a positive effect. The inclusion of interaction features significantly improved model predictions, enhancing diagnostic performance across multiple metrics.

## 4 Discussion

Our study successfully developed and validated a combined model integrating grayscale ultrasound-based radiomics features from intratumoral and peritumoral regions with clinical parameters, particularly tumor size, to predict MVI in HCC patients. By combining these three components, the model achieved superior diagnostic performance, highlighting the complementary nature of tumor size, intratumoral, and peritumoral radiomics features.

Radiomics features provide a quantitative approach to extracting high-dimensional data from medical images, enabling the characterization of tumor properties that are imperceptible to the human eye (9, 19). In our study, the majority of selected features from both intratumoral and peritumoral regions were wavelet-filtered, underscoring the importance of multi-scale texture analysis in capturing subtle variations associated with MVI. Wavelet filters decompose imaging data into frequency components, allowing the analysis of textures and patterns at different scales and orientations (20, 21). This approach is particularly effective in detecting fine structural details and subtle heterogeneities, which are often linked to biological processes such as tumor invasion and microvascular involvement (22). Previous studies have highlighted the utility of wavelet-based radiomics in enhancing predictive models for various cancers, demonstrating its robustness and versatility in capturing complex imaging features (23). The modeling results in our study demonstrated that LR and RF consistently outperformed other algorithms in both the intratumoral and peritumoral models, achieving the best performance across evaluation metrics. LR is well-suited for linearly separable data, offering simplicity and interpretability (24), while RF excels in handling non-linear relationships and interactions, reducing the risk of overfitting through ensemble learning (25). The strong performance of these algorithms in our models further validates the utility of wavelet-filtered features in predictive modeling and their ability to capture complex imaging patterns associated with MVI.

The intratumoral and peritumoral models both achieved relatively high AUC values, and no statistically significant differences were observed between them. This finding suggests that both intratumoral and peritumoral tissues contain comparable diagnostic information for MVI prediction, consistent with previous studies that have reported similar diagnostic efficacy for tumor and peritumoral features in radiomics-based models (26, 27). It also highlights the significance of peritumoral analysis, which captures interactions between the tumor and its surrounding microenvironment, providing insights complementary to intratumoral characteristics (18, 28, 29). In particular, peritumoral heterogeneity may reflect the presence of microscopic vascular remodeling, immune cell infiltration, and stromal reactions induced by tumor aggressiveness. These peritumoral changes, although not directly visible on conventional imaging, can be captured through radiomic texture analysis. Prior studies have shown that the peritumoral zone is a common site of microvascular invasion and angiogenesis, suggesting that heterogeneity in this region may indirectly indicate vascular infiltration or early metastatic spread (27). Thus, peritumoral features may serve as a surrogate marker of MVI-related microenvironmental alterations, supporting their inclusion in predictive modeling.

The combined model exhibited the highest AUC and the best performance across all evaluation metrics, underscoring its ability to integrate complementary information from tumor size, intratumoral, and peritumoral features. The results suggest that each component provides unique and synergistic contributions, collectively enhancing the model’s predictive performance. Although statistical comparisons between models did not yield significant p-values, the combined model consistently achieved higher AUC, specificity, precision, and F1-score compared to standalone models. These improvements, though not statistically significant, may still hold practical and clinical relevance in settings where improved diagnostic balance and robustness are required. Tumor size emerged as the most critical single predictive factor in the combined model. Its significance aligns with previous studies linking larger tumors to increased aggressiveness and MVI risk (30–32). However, while the clinical model achieved a relatively high AUC, its performance on other metrics was inferior to the radiomics-based models. This disparity likely reflects the limited scope of clinical parameters, which lack the nuanced and multi-dimensional data captured by radiomics features (32). The radiomics models, by integrating high-dimensional features extracted from tumor and peritumoral regions, effectively capture microstructural and textural heterogeneities associated with MVI, providing superior diagnostic performance (33).

Interestingly, tumor size demonstrated contrasting contributions in the combined model; it had the smallest coefficient in logistic regression analysis but the highest importance in SHAP analysis. This discrepancy suggests that tumor size exhibits strong interactions with intratumoral and peritumoral features, and these interactions play a significant role in enhancing the model’s predictive performance (34). Interaction analyses highlighted the synergistic effects among features, revealing complex, non-linear relationships where the combined effects exceeded the sum of individual contributions (35). These interactions enhanced diagnostic precision, model robustness, and biological interpretability (36). In our study, the probability of positive class predictions significantly increased when tumor size, intratumoral features, and peritumoral features simultaneously reached high values. This finding highlights the importance of feature interactions, particularly the interplay between intratumoral and peritumoral features combined with tumor size, in enhancing predictive performance. The combination of tumor size with radiomics features provided deeper insights into the tumor’s biological behavior, suggesting potential links between microenvironmental characteristics and structural changes captured by radiomics (37). Such findings emphasize the need for multi-feature integration to comprehensively characterize the multifaceted nature of MVI, ultimately advancing the diagnostic and prognostic capabilities of predictive models.

Although the combined model demonstrated excellent discrimination (AUC = 0.903) and overall good calibration performance, slight overestimation was observed in a limited mid-range of predicted probabilities. This is reflected in a Brier score of 0.151, indicating modest deviation from ideal probabilistic accuracy. Such discrepancies are not uncommon in binary classification tasks and may be attributed to several factors (38). First, the dataset exhibited class imbalance, with only 36 of 119 cases being MVI-positive, potentially biasing the probability distribution even after SMOTE correction. Second, the relatively small sample size, when combined with high-dimensional radiomics features, increases the risk of overfitting and suboptimal calibration. Third, while SMOTE improves training stability, it may alter the intrinsic feature distribution, affecting the reliability of predicted probabilities (39). Lastly, logistic regression models—while effective for class separation—are not inherently optimized for probabilistic calibration (40). Despite these limitations, the model’s calibration remains clinically acceptable and informative, as evidenced by the close alignment of most points with the ideal curve and a non-significant Hosmer–Lemeshow test (χ² = 11.45, p = 0.1773). Future validation using larger, multicenter datasets is warranted to further improve the model’s probabilistic reliability.

Several limitations should be acknowledged. First, this was a single-center retrospective study with a modest sample size, particularly in the MVI-positive subgroup (n = 36), which may limit the generalizability of the findings. Although SMOTE was employed to balance class distribution, its use in small-sample, high-dimensional radiomics settings may carry a risk of overfitting. To mitigate this, we adopted multiple robustness strategies including cross-validation and an independent internal validation cohort. Additionally, while the model demonstrated strong discrimination, we acknowledge the lack of external validation and prospective confirmation. A multicenter study is currently underway to address these issues and evaluate model performance across diverse clinical environments. Second, ultrasound images were obtained from different machines. Although normalization was applied to reduce variability, we cannot entirely rule out the potential influence of device-related differences on radiomics features. Third, the retrospective design inherently carries a risk of selection bias. Fourth, although MVI can be further stratified into M0, M1, and M2 grades, we treated MVI as a binary classification problem due to limited sample size. Future studies with larger, balanced cohorts are needed to explore fine-grained, multi-class prediction of MVI severity. Finally, the contribution of feature interactions, while significant, requires further validation through molecular biology experiments and independent clinical datasets. Future studies should focus on larger, multicenter cohorts and explore the integration of molecular and multimodal imaging data to enhance model performance and generalizability.

## 5 Conclusion

This study highlights the feasibility and effectiveness of integrating radiomics features from intratumoral and peritumoral regions with clinical parameters for MVI prediction in HCC. The combined model’s superior performance underscores the potential of this approach in personalized treatment planning, offering valuable insights for advancing radiomics research and its clinical applications in oncology.

## Data Availability

The original contributions presented in the study are included in the article/**Supplementary Material**. Further inquiries can be directed to the corresponding author.

## References

[1] EASL Clinical Practice Guidelines on the management of hepatocellular carcinoma. J Hepatol. (2024). doi: 10.1016/j.jhep.2024.08.028, PMID: 39690085

[2] Barcena-Varela M Monga SP Lujambio A. Precision models in hepatocellular carcinoma. Nat Rev Gastroenterol Hepatol. (2024). doi: 10.1038/s41575-024-01024-w, PMID: 39663463

[3] Hwang YJ Bae JS Lee Y Hur BY Lee DH Kim H. Classification of microvascular invasion of hepatocellular carcinoma: correlation with prognosis and magnetic resonance imaging. Clin Mol Hepatol. (2023) 29:733–46. doi: 10.3350/cmh.2023.0034, PMID: 37157775 PMC10366800

[4] Kim NR Bae H Hwang HS Han DH Kim KS Choi JS. Preoperative prediction of microvascular invasion with gadoxetic acid-enhanced magnetic resonance imaging in patients with single hepatocellular carcinoma: the implication of surgical decision on the extent of liver resection. Liver Cancer. (2024) 13:181–92. doi: 10.1159/000531786, PMID: 38751555 PMC11095589

[5] Ma L Zhang C Wen Y Xing K Li S Geng Z. Imaging-based surrogate classification for risk stratification of hepatocellular carcinoma with microvascular invasion to adjuvant hepatic arterial infusion chemotherapy: a multicenter retrospective study. Int J Surg (London England). (2024). doi: 10.1097/JS9.0000000000001903, PMID: 39051653 PMC11745592

[6] Ma X Wei J Gu D Zhu Y Feng B Liang M. Preoperative radiomics nomogram for microvascular invasion prediction in hepatocellular carcinoma using contrast-enhanced CT. Eur Radiol. (2019) 29:3595–605. doi: 10.1007/s00330-018-5985-y, PMID: 30770969

[7] Min JH Lee MW Park HS Lee DH Park HJ Lim S. Interobserver variability and diagnostic performance of gadoxetic acid-enhanced MRI for predicting microvascular invasion in hepatocellular carcinoma. Radiology. (2020) 297:573–81. doi: 10.1148/radiol.2020201940, PMID: 32990512

[8] Hong SB Choi SH Kim SY Shim JH Lee SS Byun JH. MRI features for predicting microvascular invasion of hepatocellular carcinoma: A systematic review and meta-analysis. Liver Cancer. (2021) 10. doi: 10.1159/000513704, PMID: 33981625 PMC8077694

[9] Lambin P Rios-Velazquez E Leijenaar R Carvalho S van Stiphout RGPM Granton P. Radiomics: extracting more information from medical images using advanced feature analysis. Eur J Cancer (Oxford England: 1990). (2012) 48:441–6. doi: 10.1016/j.ejca.2011.11.036, PMID: 22257792 PMC4533986

[10] Zeng S Wang X-L Yang H. Radiomics and radiogenomics: extracting more information from medical images for the diagnosis and prognostic prediction of ovarian cancer. Military Med Res. (2024) 11:77. doi: 10.1186/s40779-024-00580-1, PMID: 39673071 PMC11645790

[11] Ma X Qian X Wang Q Zhang Y Zong R Zhang J. Radiomics nomogram based on optimal VOI of multi-sequence MRI for predicting microvascular invasion in intrahepatic cholangiocarcinoma. La Radiologia Med. (2023) 128:1296–309. doi: 10.1007/s11547-023-01704-8, PMID: 37679641 PMC10620280

[12] Zhang X Ruan S Xiao W Shao J Tian W Liu W. Contrast-enhanced CT radiomics for preoperative evaluation of microvascular invasion in hepatocellular carcinoma: A two-center study. Clin Trans Med. (2020) 10:e111. doi: 10.1002/ctm2.111, PMID: 32567245 PMC7403665

[13] Yang J Dong X Jin S Wang S Wang Y Zhang L. Radiomics model of dynamic contrast-enhanced MRI for evaluating vessels encapsulating tumor clusters and microvascular invasion in hepatocellular carcinoma. Acad Radiol. (2024). doi: 10.1016/j.acra.2024.07.007, PMID: 39025700

[14] Dong Y Zuo D Qiu Y-J Cao J-Y Wang H-Z Yu L-Y. Preoperative prediction of microvascular invasion (MVI) in hepatocellular carcinoma based on kupffer phase radiomics features of sonazoid contrast-enhanced ultrasound (SCEUS): A prospective study. Clin Hemorheology Microcirculation. (2022) 81. doi: 10.3233/CH-211363, PMID: 35001883

[15] Yang Y Fan W Gu T Yu L Chen H Lv Y. Radiomic features of multi-ROI and multi-phase MRI for the prediction of microvascular invasion in solitary hepatocellular carcinoma. Front Oncol. (2021) 11:756216. doi: 10.3389/fonc.2021.756216, PMID: 34692547 PMC8529277

[16] Gao L Xiong M Chen X Han Z Yan C Ye R. Multi-region radiomic analysis based on multi-sequence MRI can preoperatively predict microvascular invasion in hepatocellular carcinoma. Front Oncol. (2022) 12:818681. doi: 10.3389/fonc.2022.818681, PMID: 35574328 PMC9094629

[17] Yushkevich PA Piven J Hazlett HC Smith RG Ho S Gee JC. User-guided 3D active contour segmentation of anatomical structures: significantly improved efficiency and reliability. NeuroImage. (2006) 31:1116–28. doi: 10.1016/j.neuroimage.2006.01.015, PMID: 16545965

[18] Huang Y Qian H. Advancing hepatocellular carcinoma management through peritumoral radiomics: enhancing diagnosis, treatment, and prognosis. J Hepatocellular Carcinoma. (2024) 11:2159–68. doi: 10.2147/JHC.S493227, PMID: 39525830 PMC11546143

[19] Lambin P Leijenaar RTH Deist TM Peerlings J de Jong EEC van Timmeren J. Radiomics: the bridge between medical imaging and personalized medicine. Nat Rev Clin Oncol. (2017) 14:749–62. doi: 10.1038/nrclinonc.2017.141, PMID: 28975929

[20] Zhou J Lu J Gao C Zeng J Zhou C Lai X. Predicting the response to neoadjuvant chemotherapy for breast cancer: wavelet transforming radiomics in MRI. BMC Cancer. (2020) 20:100. doi: 10.1186/s12885-020-6523-2, PMID: 32024483 PMC7003343

[21] Jiang Z Yin J Han P Chen N Kang Q Qiu Y. Wavelet transformation can enhance computed tomography texture features: a multicenter radiomics study for grade assessment of COVID-19 pulmonary lesions. Quantitative Imaging In Med Surg. (2022) 12:4758–70. doi: 10.21037/qims-22-252, PMID: 36185061 PMC9511418

[22] Abd El Kader I Xu G Shuai Z Saminu S Javaid I Ahmad IS. Brain tumor detection and classification on MR images by a deep wavelet auto-encoder model. Diagnostics (Basel Switzerland). (2021) 11. doi: 10.3390/diagnostics11091589, PMID: 34573931 PMC8471235

[23] Ginsburg SB Algohary A Pahwa S Gulani V Ponsky L Aronen HJ. Radiomic features for prostate cancer detection on MRI differ between the transition and peripheral zones: Preliminary findings from a multi-institutional study. J Magnetic Resonance Imaging: JMRI. (2017) 46:184–93. doi: 10.1002/jmri.25562, PMID: 27990722 PMC5464994

[24] Bertsimas D King A. Logistic regression: from art to science. Stat Sci. (2017) 32:367–84. doi: 10.1214/16-STS602

[25] Lever J Krzywinski M Altman N. Points of significance: logistic regression. Nat Methods. (2016) 13:541–2. doi: 10.1038/nmeth.3904

[26] Dong Y Zhou L Xia W Zhao X-Y Zhang Q Jian J-M. Preoperative prediction of microvascular invasion in hepatocellular carcinoma: initial application of a radiomic algorithm based on grayscale ultrasound images. Front Oncol. (2020) 10:353. doi: 10.3389/fonc.2020.00353, PMID: 32266138 PMC7096379

[27] Nebbia G Zhang Q Arefan D Zhao X Wu S. Pre-operative microvascular invasion prediction using multi-parametric liver MRI radiomics. J Digital Imaging. (2020) 33:1376–86. doi: 10.1007/s10278-020-00353-x, PMID: 32495126 PMC7728938

[28] Qian H Shen Z Zhou D Huang Y. Intratumoral and peritumoral radiomics model based on abdominal ultrasound for predicting Ki-67 expression in patients with hepatocellular cancer. Front Oncol. (2023) 13:1209111. doi: 10.3389/fonc.2023.1209111, PMID: 37711208 PMC10498123

[29] Qian H Huang Y Xu L Fu H Lu B. Role of peritumoral tissue analysis in predicting characteristics of hepatocellular carcinoma using ultrasound-based radiomics. Sci Rep. (2024) 14:11538. doi: 10.1038/s41598-024-62457-6, PMID: 38773179 PMC11109225

[30] Esnaola N Lauwers G Mirza N Nagorney D Doherty D Ikai I. Predictors of microvascular invasion in patients with hepatocellular carcinoma who are candidates for orthotopic liver transplantation. J Gastrointestinal Surg. (2002) 6:224–32. doi: 10.1016/S1091-255X(01)00015-4, PMID: 11992808

[31] Hu H Qi S Zeng S Zhang P He L-Y Wen S. Importance of microvascular invasion risk and tumor size on recurrence and survival of hepatocellular carcinoma after anatomical resection and non-anatomical resection. Front Oncol. (2021) 11:621622. doi: 10.3389/fonc.2021.621622, PMID: 33816254 PMC8010691

[32] Xu X Zhang H-L Liu Q Sun S Zhang J Zhu F. Radiomic analysis of contrast-enhanced CT predicts microvascular invasion and outcome in hepatocellular carcinoma. J Hepatol. (2019) 70:1133–44. doi: 10.1016/j.jhep.2019.02.023, PMID: 30876945

[33] Yang L Gu D Wei J Yang C Rao S Wang W. A radiomics nomogram for preoperative prediction of microvascular invasion in hepatocellular carcinoma. Liver Cancer. (2019) 8:373–86. doi: 10.1159/000494099, PMID: 31768346 PMC6873064

[34] Usset J Staicu A-M Maity A. Interaction models for functional regression. Comput Stat Data Anal. (2016) 94:317–29. doi: 10.1016/j.csda.2015.08.020, PMID: 26744549 PMC4698914

[35] Luo R Qi X. Interaction model and model selection for function-on-function regression. J Comput Graphical Stat. (2019) 28:309–22. doi: 10.1080/10618600.2018.1514310

[36] Grossmann P Stringfield O El-Hachem N Bui MM Rios Velazquez E Parmar C. Defining the biological basis of radiomic phenotypes in lung cancer. ELife. (2017) 6. doi: 10.7554/eLife.23421, PMID: 28731408 PMC5590809

[37] La Greca Saint-Esteven A Vuong D Tschanz F van Timmeren JE Dal Bello R Waller V. Systematic review on the association of radiomics with tumor biological endpoints. Cancers. (2021) 13. doi: 10.3390/cancers13123015, PMID: 34208595 PMC8234501

[38] Niculescu-Mizil A Caruana R. (2005). Predicting good probabilities with supervised learning, in: Machine Learning, Proceedings of the Twenty-Second International Conference (ICML 2005), Association for Computing Machinery (ACM), New York, NY, USA.

[39] Fernández A García S Herrera F Chawla NV. SMOTE for learning from imbalanced data: progress and challenges, marking the 15-year anniversary. J Artif Int Res. (2018) 61:863–905. doi: 10.1613/jair.1.11192

[40] Zadrozny B Elkan C. Transforming classifier scores into accurate multiclass probability estimates. In: Proceedings of the eighth ACM SIGKDD international conference on Knowledge discovery and data mining. Association for Computing Machinery, Edmonton, Alberta, Canada (2002). doi: 10.1145/775047.775151

