# Supplementary material for: Integrating intratumoral, peritumoral, and clinical features in an ultrasound-based radiomics model: contributions and synergies for predicting microvascular invasion in hepatocellular carcinoma
Source: Front Oncol. 2025 Sep 1;15:1566105. doi: 10.3389/fonc.2025.1566105 (PMC12433787; doi:10.3389/fonc.2025.1566105)
Supplement: Supplementary file 1 [file DataSheet1.docx]

1. **Ultrasound machines**

Ultrasound examination was performed by using one of the following ultrasound machines: LOGIQ E8 (GE Healthcare, United States; C5-1 convex array probes, 1–5 MHz); LOGIQ E9 (GE Healthcare, United States; C5-1 convex array probes, 1–5 MHz); Aplio 500 (Toshiba Medical systems, Japan; 6C1 probe, 1–6 MHz); i800 (Cannon Medical systems Corporation, Japan; i8CX1 probe, 1-8MHz); and Resona 7T (Mindray, China; SC6-1 U probe, 1-6MHz).

**Tables**

**Table S1 Comparison of clinical characteristics between the training and validation groups**

| **Variables** | **Training Group(n=83)** | **Testing Group((n=36)** | **p** |  |
| --- | --- | --- | --- | --- |
|  |  |  |  |  |
| **Age(year)** | 65.86±10.48 | 62.64±10.79 | 0.134 |  |
| **AFP(mg/mL)** | 12.66(3.58-86.76) | 9.66(3.88-81.69) | 0.147 |  |
| **ALT(IU/L)** | 41.32±41.44 | 34.59±24.2 | 0.369 |  |
| **AST(IU/L)** | 48.6±50.23 | 35.65±18.69 | 0.139 |  |
| **TBIL(µmol/L)** | 16.42±10.42 | 20.24±25.52 | 0.253 |  |
| **DBIL(µmol/L)** | 5.72±5.22 | 7.52±14.92 | 0.338 |  |
| **ALB (g/L)** | 38.56±4.25 | 39.3±4.3 | 0.395 |  |
| **PT(s)** | 13.03±1.17 | 13.33±1.68 | 0.261 |  |
| **INR** | 1.04±0.1 | 1.07±0.16 | 0.29 |  |
| **Tumor Size** | 4.75±2.77 | 5.25±2.93 | 0.383 |  |
| **Sex** |  |  | 0.166 |  |
| Female | 21 | 5 |  |  |
| Male | 62 | 31 |  |  |
| **HBsAg** |  |  | 0.699 |  |
| Negative | 26 | 10 |  |  |
| Positive | 57 | 26 |  |  |
| **Cirrhosis** |  |  | 0.221 |  |
| Absent | 36 | 20 |  |  |
| Present | 47 | 16 |  |  |
| **Multifocality** |  |  | 0.389 |  |
| Absent | 70 | 28 |  |  |
| Present | 13 | 8 |  |  |

AFP, alpha fetoprotein; ALB, albumin level; ALT, alanine aminotransferase; AST, aspartate aminotransferase; TBIL, total bilirubin; DBIL, directed bilirubin; PT, prothrombin time; INR, international normalized ratio.

**Table S2 Hyperparameter Search Ranges**

| **Model** | **Parameter** | **Search Range** |
| --- | --- | --- |
| **Support Vector Machine** | C | 0.001, 0.01, 0.1, 1, 10, 100, 1000, 10000 |
|  | kernel | linear, rbf |
|  | gamma | 0.001, 0.01, 0.1, 1, 10, 100, 1000, 10000 |
| **Random Forest** | n_estimators | 100, 200, 300 |
|  | max_depth | None, 10, 20, 30 |
|  | min_samples_split | 2, 5, 10 |
|  | min_samples_leaf | 1, 2, 4 |
| **K-Nearest Neighbors** | n_neighbors | 1-30 |
|  | weights | uniform, distance |
|  | p | 1 (Manhattan), 2 (Euclidean) |
| **Logistic Regression** | C | 0.001-1000 |
|  | penalty | l1, l2 |
| **Decision Tree** | max_depth | None, 10, 20, 30 |
|  | min_samples_split | 2, 5, 10 |
|  | min_samples_leaf | 1, 2, 4 |
| **MLP Classifier** | hidden_layer_sizes | (100,), (100, 50), (100, 100), (50, 50), (30, 20, 10) |
|  | activation | relu, tanh, logistic |
|  | alpha | 1e-6, 1e-5, 1e-4, 1e-3, 1e-2 |
|  | learning_rate | constant, invscaling, adaptive |
| **AdaBoost Classifier** | n_estimators | 50, 100, 200 |
|  | learning_rate | 0.001-1 |
| **Gradient Boosting** | n_estimators | 50, 100, 200 |
|  | learning_rate | 0.001-1 |
|  | max_depth | None, 3, 5, 7, 9 |
| **XGBoost** | n_estimators | 50, 100, 200 |
|  | learning_rate | 0.001-1 |
|  | max_depth | 3, 5, 7 |
|  | gamma | 0-5 |

**Table S3 Best parameters in the intratumoral radiomics model**

| **Model** | **Best Parameters** |
| --- | --- |
| **Support Vector Machine** | {'kernel': 'linear', 'gamma': 100.0, 'C': 1000.0} |
| **RandomForest** | {'n_estimators': 300, 'min_samples_split': 2, 'min_samples_leaf': 1, 'max_depth': 30} |
| **K-Nearest Neighbor** | {'weights': 'uniform', 'p': 1, 'n_neighbors': 1} |
| **LogisticRegression** | {'penalty': 'l2', 'C': 10.0} |
| **DecisionTree** | {'min_samples_split': 2, 'min_samples_leaf': 2, 'max_depth': 10} |
| **MLPClassifier** | {'learning_rate': 'constant', 'hidden_layer_sizes': (30, 20, 10), 'alpha': 0.01, 'activation': 'relu'} |
| **AdaBoostClassifier** | {'n_estimators': 50, 'learning_rate': 0.001} |
| **GradientBoostingClassifier** | {'n_estimators': 100, 'max_depth': 7, 'learning_rate': 0.01} |
| **XGBOOST** | {'n_estimators': 200, 'max_depth': 7, 'learning_rate': 0.1, 'gamma': 0.1} |

**Table S4 Best parameters in the peritumoral radiomics model**

| **Model** | **Best Parameters** |
| --- | --- |
| **Support Vector Machine** | {'kernel': 'linear', 'gamma': 0.001, 'C': 1.0} |
| **RandomForest** | {'n_estimators': 300, 'min_samples_split': 2, 'min_samples_leaf': 1, 'max_depth': 30} |
| **K-Nearest Neighbor** | {'weights': 'uniform', 'p': 1, 'n_neighbors': 2} |
| **LogisticRegression** | {'penalty': 'l2', 'C': 0.1} |
| **DecisionTree** | {'min_samples_split': 2, 'min_samples_leaf': 2, 'max_depth': 20} |
| **MLPClassifier** | {'learning_rate': 'invscaling', 'hidden_layer_sizes': (50, 50), 'alpha': 0.0001, 'activation': 'tanh'} |
| **AdaBoostClassifier** | {'n_estimators': 200, 'learning_rate': 0.1} |
| **GradientBoostingClassifier** | {'n_estimators': 50, 'max_depth': None, 'learning_rate': 0.001} |
| **XGBOOST** | {'n_estimators': 200, 'max_depth': 7, 'learning_rate': 0.1, 'gamma': 0.1} |

**Figures**

**
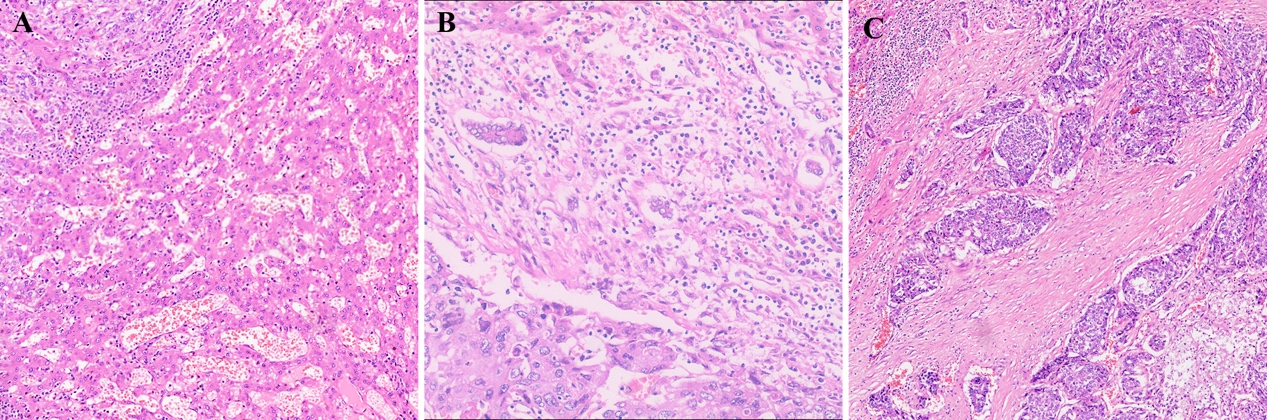
**

**Fig S1.** Representative histopathological images illustrating microvascular invasion (MVI) in hepatocellular carcinoma. (A) M0: no microvascular invasion observed; (B) M1: ≤5 MVI sites within 1 cm of the tumor margin; (C) M2: >5 MVI sites or invasion beyond 1 cm. Hematoxylin and eosin staining, ×100 magnification.

**
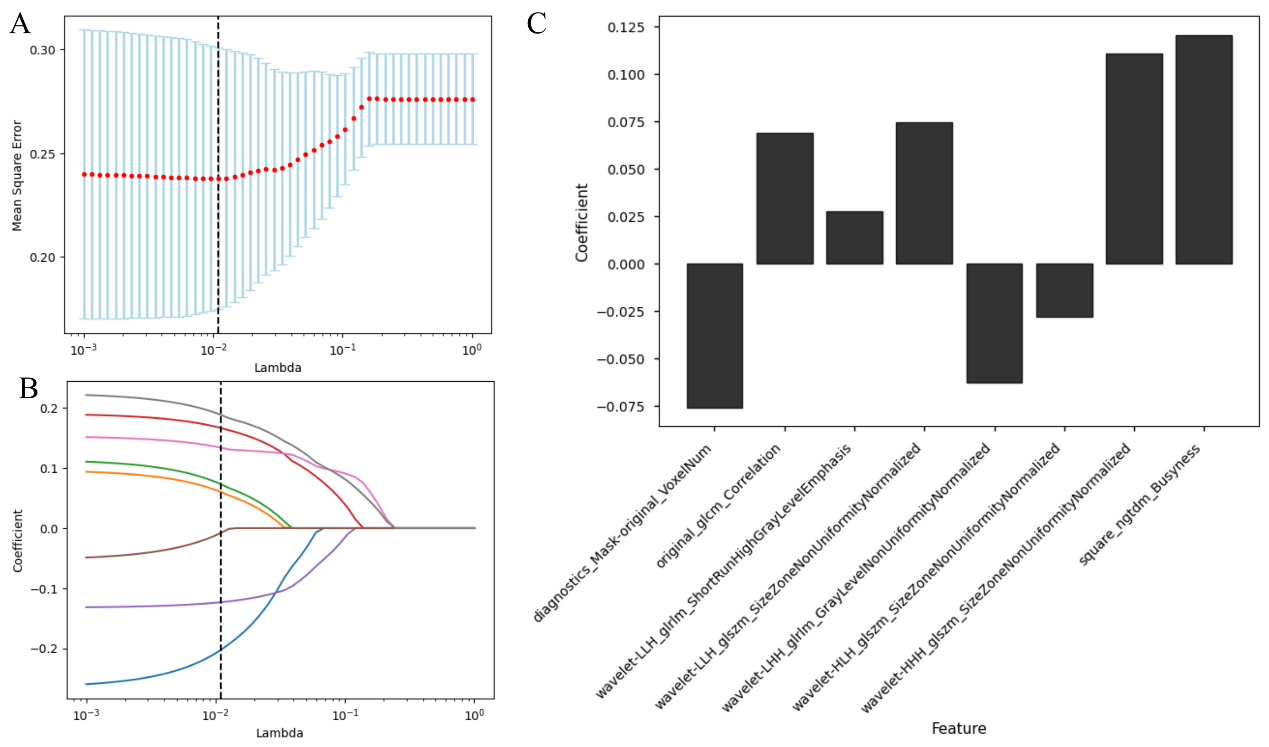
**

**Fig S2.** Intratumoral model LASSO procedure: (A, B) Least absolute shrinkage and selection operator (LASSO) regression complexity was controlled using a tuning parameter lambda; the optimal lambda value for the minimized mean squared error was 0.010985411419875584. (C) The 8 selected variables and their coefficient values are shown.

**
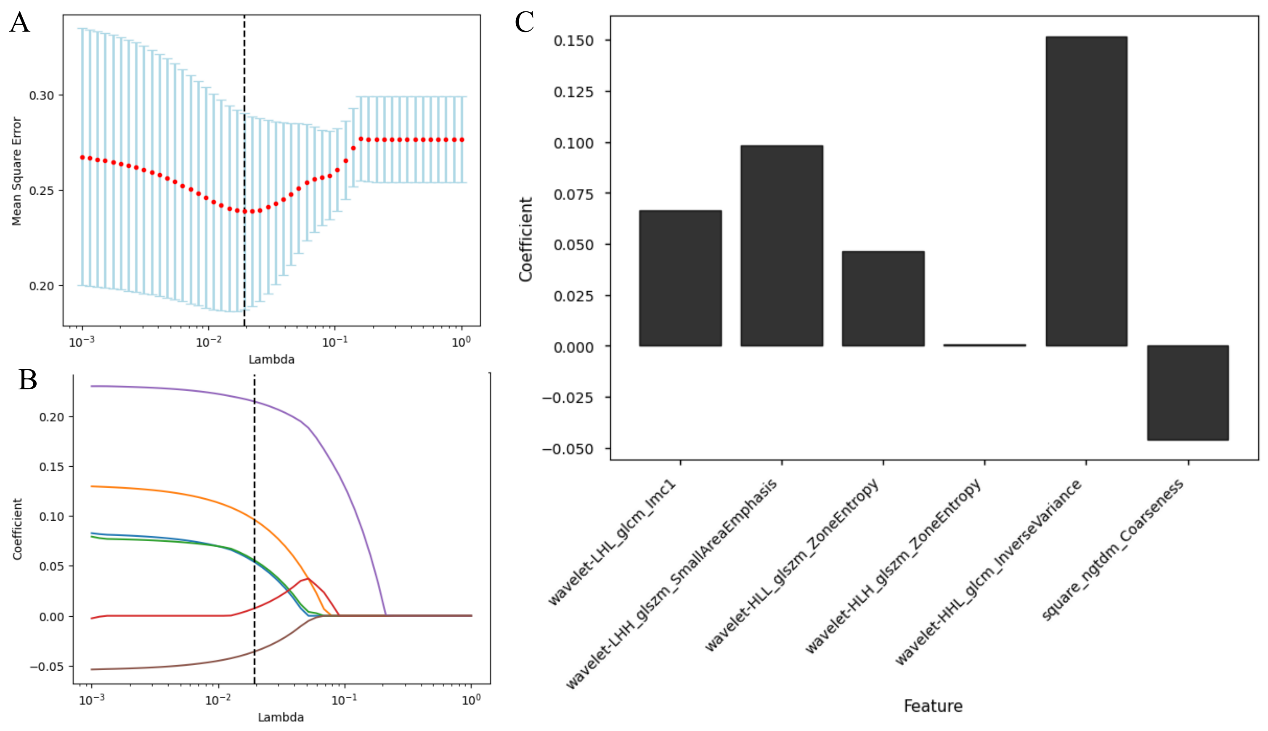
**

**Fig S3.** Peritumoral model LASSO procedure: (A, B) Least absolute shrinkage and selection operator (LASSO) regression complexity was controlled using a tuning parameter lambda; the optimal lambda value for the minimized mean squared error was 0.019306977288832496. (C) The 6 selected variables and their coefficient values are shown.


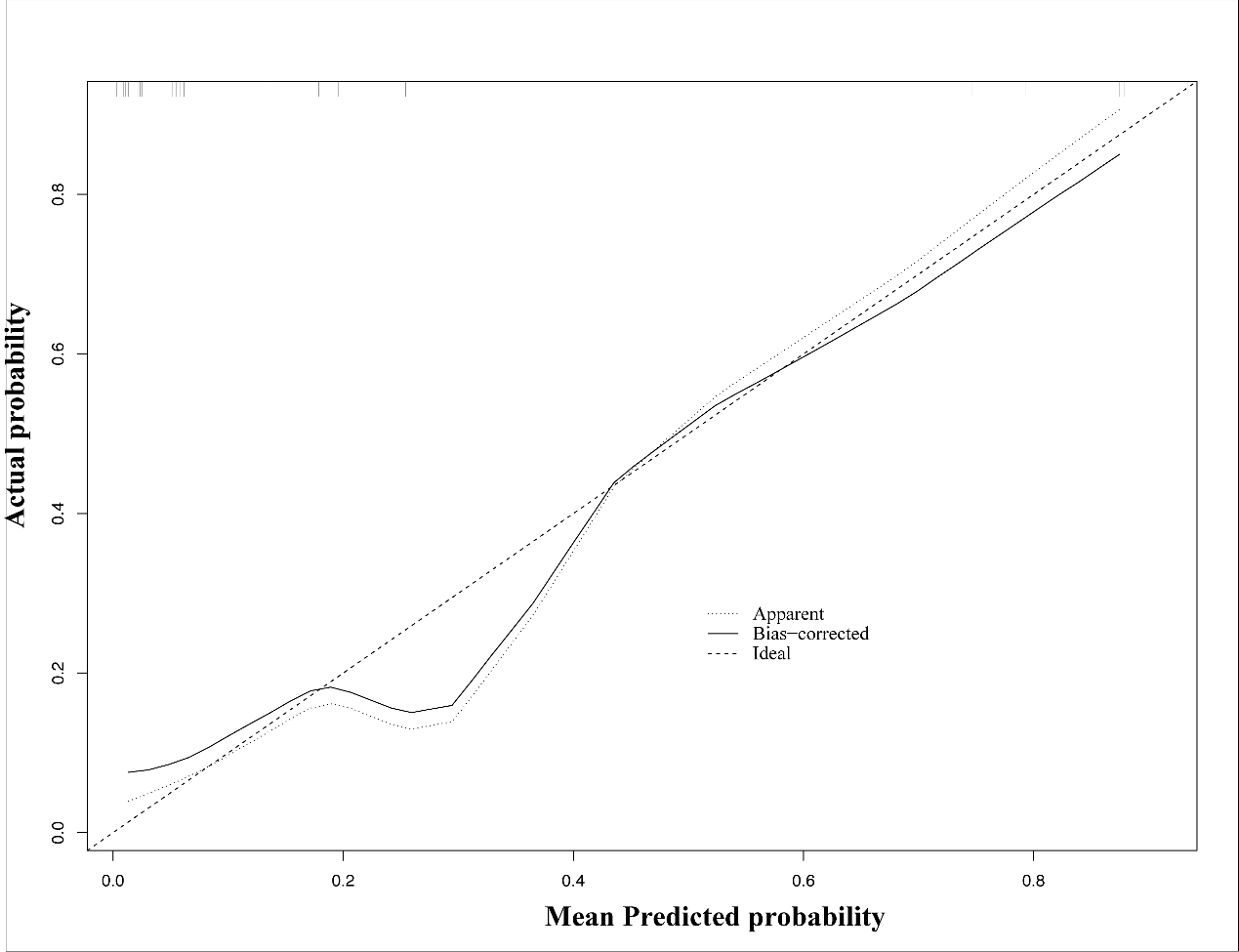


**Fig S4.** Calibration curve of the combined model in the validation cohort. The x-axis represents the predicted probability of microvascular invasion (MVI), and the y-axis represents the observed proportion of MVI-positive cases. The gray dashed diagonal line denotes perfect calibration. The combined model shows overall good calibration, with only mild deviations observed in a limited probability range. The Hosmer–Lemeshow test yielded a non-significant result (χ² = 11.45, p = 0.1773), indicating no evidence of poor model fit.
